# Supplementary material for: Confidence intervals for the difference between the coefficients of variation of Weibull distributions for analyzing wind speed dispersion
Source: PeerJ. 2021 Jul 2;9:e11676. doi: 10.7717/peerj.11676 (PMC8256813; doi:10.7717/peerj.11676)
Supplement: Supplemental Information 1 [file peerj-09-11676-s001.docx]

Dataset 1 : The wind speeds measured at 90-meter wind energy potential stations in Trad province and Chonburi province in 2016

| **Trad** | **Chonburi** |
| --- | --- |
| 2.49 | 4.79 |
| 3.47 | 5.15 |
| 2.45 | 4.91 |
| 2.50 | 4.49 |
| 1.70 | 4.09 |
| 2.15 | 4.89 |
| 1.35 | 3.59 |
| 1.74 | 3.38 |
| 0.33 | 3.47 |
| 0.37 | 4.55 |
| 0.32 | 4.33 |
| 0.32 |  |

Source: Department of Alternative Energy Development and Efficiency, ministry of energy.
