# Supplementary material for: Confidence intervals for the difference between the coefficients of variation of Weibull distributions for analyzing wind speed dispersion
Source: PeerJ. 2021 Jul 2;9:e11676. doi: 10.7717/peerj.11676 (PMC8256813; doi:10.7717/peerj.11676)
Supplement: Supplemental Information 2 — Collected April-May 2019. [file peerj-09-11676-s002.docx]

Dataset 2 : The wind speeds measured at 90-meter wind energy potential stations in the southern and northeastern regions of Thailand were collected in April-May 2019.

| **Southern** | **Northeastern** |
| --- | --- |
| 3.79 | 2.63 |
| 0.41 | 5.62 |
| 1.88 | 5.96 |
| 3.49 | 3.71 |
| 2.37 | 5.57 |
| 3.13 | 4.54 |
| 3.56 | 4.53 |
| 1.15 | 4.59 |
| 1.59 | 1.98 |
| 1.98 | 3.72 |
| 1.73 | 1.53 |
| 1.92 | 4.10 |
|  | 2.33 |
|  | 4.89 |
|  | 4.60 |
|  | 2.77 |
|  | 4.93 |
|  | 5.19 |
|  | 4.30 |
|  | 3.13 |

Source: Department of Alternative Energy Development and Efficiency, ministry of energy.
